# Detection and Localization of Early-Stage Multiple Brain Tumors Using a Hybrid Technique of Patch-Based Processing, k-means Clustering and Object Counting

**DOI:** 10.1155/2020/9035096

**Published:** 2020-01-06

**Authors:** Mohamed Nasor, Walid Obaid

**Affiliations:** Department of Biomedical Engineering, Ajman University, Ajman, UAE

## Abstract

Brain tumors are a major health problem that affect the lives of many people. These tumors are classified as benign or cancerous. The latter can be fatal if not properly diagnosed and treated. Therefore, the diagnosis of brain tumors at the early stages of their development can significantly improve the chances of patient's full recovery after treatment. In addition to laboratory analyses, clinicians and surgeons extract information from medical images, recorded by various systems such as magnetic resonance imaging (MRI), X-ray, and computed tomography (CT). The extracted information is used to identify the essential characteristics of brain tumors (location, size, and type) in order to achieve an accurate diagnosis to determine the most appropriate treatment protocol. In this paper, we present an automated machine vision technique for the detection and localization of brain tumors in MRI images at their very early stages using a combination of *k*-means clustering, patch-based image processing, object counting, and tumor evaluation. The technique was tested on twenty real MRI images and was found to be capable of detecting multiple tumors in MRI images regardless of their intensity level variations, size, and location including those with very small sizes. In addition to its use for diagnosis, the technique can be integrated into automated treatment instruments and robotic surgery systems.

## 1. Introduction

A brain tumor is an abnormal mass of tissues that grows and multiplies rapidly. It can originate within the brain, which is called primary brain tumor, or in other surrounding areas and moves to the brain at a later stage, which is called metastatic brain tumor. There are more than 120 types of tumors classified as benign or malignant (cancerous). Cancerous tumors cause serious health problems such as severe headaches, blindness, and paralysis [1]. The timely detection and diagnosis of these tumors at their very early stages help clinicians to decide the most appropriate treatment protocol.

Magnetic resonance imaging (MRI) is one of the most advanced medical imaging modalities. It is a noninvasive soft tissue contrast imaging used for the diagnosis of tumors within the human brain tissues [2]. MRI system produces image scans through the application of a combination of radio waves and a strong magnetic field to align the magnetic spins in body organs along the magnetic field. When the radio frequency is turned off, the spin system produces a signal, called the free induction decay (FID) signal, which reflects the water content of brain tissues. The FID signal is processed further to give a two dimensional (2D) image of the organ tissues. MRI systems are capable of producing images of different sections (slices) within the brain with no overlap of other anatomical structures, which can provide detailed information about brain tumors such as exact location, shape, and size. This information can help clinicians and surgeons to reach an accurate diagnosis of tumors in order to determine the appropriate treatment procedure/protocol such as surgery, chemotherapy, and radiotherapy [3]. Manual extraction of the essential clinical information from the MRI images is not an easy task because of the complex nature of these scans, which requires interpretation by skilled and experienced medical professionals. Moreover, the large number of MRI images recorded in hospitals and clinics, makes manual segmentation a very tedious and time-consuming task. To accelerate the diagnosis process and make it accurate and reliable, various automated segmentation and detection techniques were developed [4].

## 2. Related Work

Brain image segmentation begins with image preprocessing which includes eliminating noncerebral tissues from the image using a process known as skull stripping, followed by intensity normalization and filtering of noisy pixels [5, 6]. Various techniques of skull stripping have been developed over the years. A skull stripping technique presented by Hahn et al. is based on a 3D watershed transformation using a combination of white matter three-dimensional connectivity and a modified watershed algorithm combined with preflooding in order to avoid oversegmentation [7]. Ségonne et al. proposed a hybrid approach of deformable surface models and watershed algorithms, which involves estimating the brain volume by three-dimensional connectivity operation [8]. Sadananthan et al. developed a skull stripping technique, which involves graph cuts, intensity thresholding, followed by removal of narrow connections to obtain a brain mask [9]. These approaches are relatively successful in determining the brain edge but they are highly complex, computationally intensive, and prone to the possibility of brain tissue erosion and oversampling. In this paper, we have implemented a fast and accurate skull stripping technique that involves multiple thresholding and object counting.

Various fully automatic medical image segmentation techniques that involve various machine vision techniques have been described in the literature [10]. A technique presented by Karkanis et al. is based on wavelet decomposition for obtaining the color wavelet covariance features of the second-order textural measures in an endoscopic video [11]. Another technique suggested by Logeswari et al., involves hierarchical self-organizing maps generated by removing noise and artifacts and then identifying the principle tissue structures using fuzzy *c*-means clustering [12]. An approach proposed by Sinha et al. for tumor detection consists of three techniques: *k*-means clustering with watershed segmentation, optimized *k*-means clustering with genetic algorithm, and optimized *c*-means clustering with genetic algorithm [13]. A technique for tumor region detection, developed by Megersa et al. combines skull stripping and fuzzy Hopfield neural networks [14]. A technique introduced by Bahadure et al. uses Berkley wavelet transformation and support vector machine to improve the segmentation process by extracting features from the segmented tissues [15]. Hanuman et al. developed a technique for brain tumor segmentation, which includes anisotropic diffusion, *k*-means clustering, morphological operations, temporal smoothing, and volumetric measurement [16]. A brain tumor detection technique proposed by Hazra et al. is comprised of three stages: noise removal, edge detection, and *k*-means clustering [17]. Kharrat et al. proposed an efficient technique for the detection of brain tumors that includes morphological operations to enhance the image contrast followed by wavelet transformation for segmentation and *k*-means clustering for extracting the tumor [18]. Gujar et al. suggested a technique for brain image segmentation that combines *k*-means clustering and genetic algorithms [19]. An automatic segmentation technique introduced by Pereira et al. consists of three steps: preprocessing, classification with convolutional neural network, and post-processing [20]. A brain tumor segmentation technique using ant colony optimization (ACO) was proposed by Kullayamma et al. [21]. A new approach to improve the segmentation accuracy of brain tumors based on temperature profiles changes in the tumorous region was proposed Bousselham et al. [22]. In this approach, Pennes bioheat equation and Canny edge detection method were used to estimate tumor contours based on the change of temperature. Brain tumor segmentation based on hybrid clustering and morphological operations was proposed by Zhang et al. [23] which consists of adaptive Wiener filtering for denoising, morphological operations to remove non cerebral tissues and *k*-means clustering combined with Gaussian kernel-based fuzzy *c*-means algorithm to segment images.

The majority of the techniques discussed above give a general segmented MRI image without localizing the tumor region; some techniques can detect a single tumor, but none of them addressed the detection and localization of multiple and very small tumors. In this paper, we propose an automated technique that can detect and localize multiple brain tumors, including those with very small sizes. The technique begins with an initialization step using *k*-means clustering to identify the brain surrounding edge, followed by dividing the MRI image into patches that are iteratively scaled, followed by object detection and counting using multiple threshold values. The novelty and contribution of the proposed technique is that it can detect both large and small tumors in the same MRI image without the need for advanced machine learning algorithms or direct comparison of the tested images with reference images from a database.

## 3. Methodology

The proposed technique involves four image processing operations: *k*-means clustering, patch-based processing, object counting and tumor evaluation, discussed in the following sections.

### 3.1. *k*-Means Clustering

*k*-Means clustering is an unsupervised clustering algorithm that divides the intensities in the image based on cluster centroids, followed by calculating the distance between each image pixel and the corresponding centroid. The algorithm assigns each pixel to a specific centroid based on the minimum distance value. The algorithm also updates the centroids by finding the average distance values of the assigned pixels of the centroids. The values of the distances are updated with respect to the new centroids and the pixels are re-assigned. The algorithm continues until there are no significant changes in the distances from the centroids [16]. Effective *k*-means clustering is achieved by minimizing the within cluster variance, which is the sum of squares within each cluster (SSW), and maximizing the between cluster variance, which is the sum of squares between clusters (SSB), as follows:

(1) $$\begin{array}{c} SSW\left(C,K\right)=\overset{N}{\underset{i=1}{\sum }}{||x_{i}-c_{k}||}^{2} \end{array}$$

(2) $$\begin{array}{c} SSB\left(C,K\right)=\overset{K}{\underset{j=1}{\sum }}n_{j}{||c_{j}-\overset{¯}{x}||}^{2} \end{array}$$

*k* = number of clusters, $\overset{¯}{x}$ = overall centroid, *N* = number of objects in a data set, {*C*1 … *Ck*} = set of clusters, *n*_*j*_ = number of objects in cluster *C*_*j*_, and *c*_*j*_ = centroid of *C*_*j*_.

**Figure 1:**
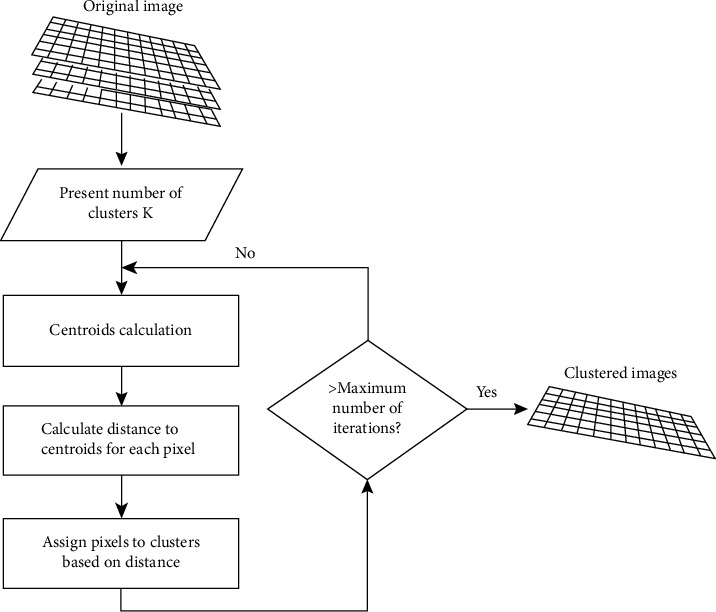
Flowchart of the *k*-means algorithm [24].

The lower the total SSW value is, the greater the intra-cluster cohesion associated with the given cluster configuration and the higher the total SSB value of a cluster configuration is, the greater the degree of separation. A flowchart of the *k*-means clustering algorithm is shown in Figure 1.

### 3.2. Patch-Based Image Processing

In patch-based image processing, the original image is divided into small patches, which are processed independently and subsequently combined to give the final processed image. In the proposed technique, the MRI image is uniformly divided into multiple patches of the original MRI image. In order to detect and localize smaller tumors which cannot be detected at the normal scale of the image, each patch is scaled up to three times its original size. Each scaled patch is processed using *k*-means, object counting, and tumor evaluation for the detection and localization of tumors in the selected patch. The processed patches are combined to give the total detected tumors in the original MRI image.

### 3.3. Tumors Detection Using Object Counting

Object counting is a technique used to detect objects in images for the purpose of localization and counting. An object is defined as a group of bright pixels that form a connected component in a binary image. Brain tuomors are considered as objects since they appear in MRI images as bright areas. In order to detect tumors the original MRI image is converted to multiple binary images using different threshold values followed by continuous erosion coupled with object counting until the number of objects in each binary image is equal to one object. The produced images are subsequently added together into one image. If the image contains more than one connected component, the connected component having pixels with maximum integer value is considered for region growing to give an object, which represents a possible tumor. On the other hand, if the image has no object, then there are no more tumors in the original image. Once a possible tumor is detected, it is eliminated from the original MRI image and the object counting process is repeated in order to detect and localize all possible tumors in the MRI image [22].

### 3.4. Tumor Evaluation

Brain tumors usually do not occur very close to the skull. They occur within the cerebral tissues of the brain, therefore the bright objects that appear adjacent to the skull are considered as false tumors. To identify and eliminate such objects, the Euclidean distance between the possible tumor and the largest surrounding edge of the skull is used. If it is found to be less than a predefined threshold, that possible tumor is considered as a false tumor. On the other hand, if the Euclidean distance is greater than the predefined threshold value, the possible tumor is considered as a true tumor in the MRI image [22].

### 3.5. The Proposed Technique Steps

The proposed technique comprises the following processing steps:

(i) Clustering the MRI image using *k*-means into three regions: bright regions (possible tumors), medium intensity regions (including cerebrum) and dark regions. Figure 1 shows a flowchart of the *k*-means clustering.

(ii) Localizing and enhancing the brain edge. Unlike skull stripping in which the surrounding edge is removed, in this technique, the surrounding edge is enhanced and used for the elimination of false tumors.

(iii) Selecting a threshold for the Euclidean distance between the brain surrounding edge and cerebral tissues to eliminate false tumors that are part of the skull.

(iv) Converting the original MRI image into two images: the first image is obtained by multiplying the medium intensity *k*-means cluster with the inverted largest surrounding edge that is expanded towards the centre, followed by selecting the largest connected component and filling the holes. The second image is obtained by filtering the original MRI image using an averaging filter with a small kernel and multiplying the filtered image by the intensity adjusted original MRI image within the range of 49.99–50.01%. Performing further adjustment of the intensities of the pixels in the MRI image using the range between 10% and 90%.

(v) Splitting the converted image into patches, which undergo scaling-up to three times their initial sizes, in order to magnify small tumors so that they can be detected in the next step.

(vi) Detecting all possible tumors in each patch by binary conversion using multiple threshold values and applying object counting with erosion until the number of objects equals one followed by region growing.

(vii) Repeating steps (v)–(vi) for the original MRI image.

(viii) Repeating steps (v)–(vii) starting from a shifted index by 50 pixels and selecting the resultant tumors which were detected at least twice in the repeated steps.

(ix) Eliminating false tumors that are close to the largest surrounding edge of the skull by applying the tumor evaluation criteria.

**Figure 2:**
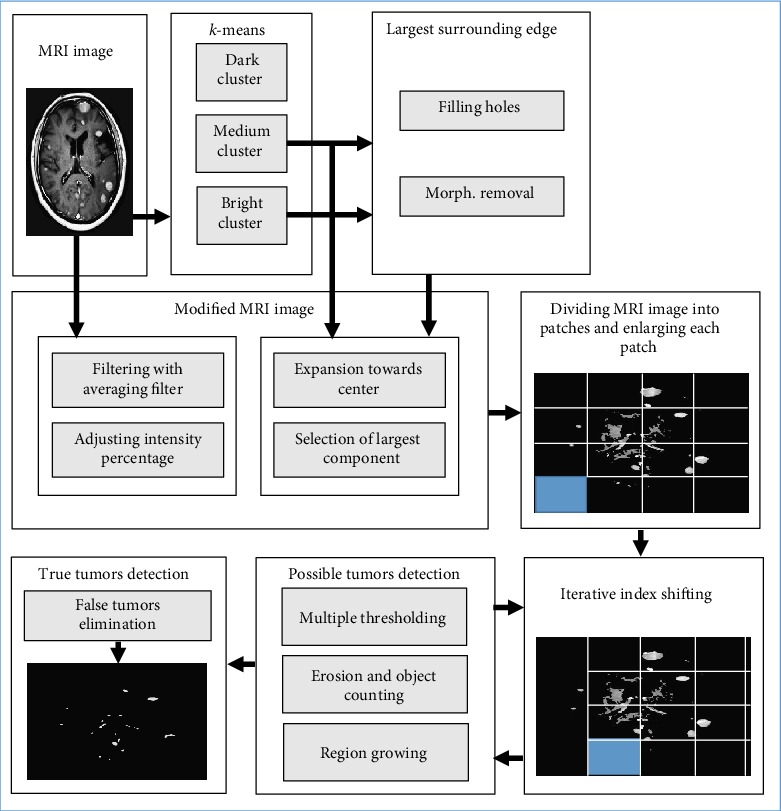
Block diagram of the proposed MRI image detection and localization technique.

A block diagram of the proposed technique is given in Figure 2.

## 4. Implementation and Results

**Figure 3:**
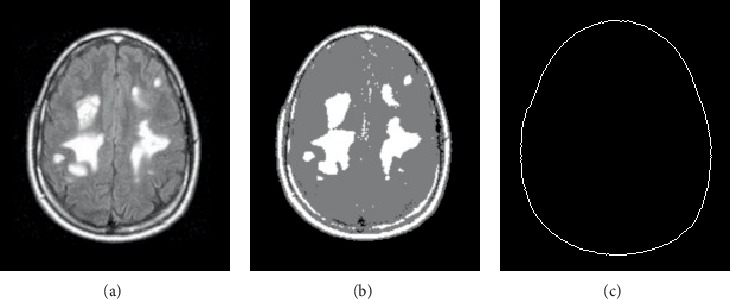
Applying *k*-means clustering on the MRI image and detection of Skull edge. (a) Original MRI image with tumors of different sizes. (b) *k*-means clustered MRI image. (c) Detection of the largest surrounding edge (skull).

The proposed technique was implemented and tested using an algorithm developed using Matlab^TM^. The technique was tested for detecting small size tumors using 20 real MRI images. The selected MRI images contained multiple early-stage tumors in addition to larger tumors as shown in Figure 3(a). The implementation started with applying *k*-means clustering for the MRI image in order to cluster the image into three main regions: bright regions that include possible tumors, medium intensity regions that include normal brain tissues and dark regions that do not include tissues or skull parts, as shown in Figure 3(b). The largest surrounding edge was determined by considering two of the *k*-means clusters: the bright regions and the medium intensity regions. Morphological removal of the interior pixels was performed to obtain an outline for the boundaries of both clusters. This process resulted in detecting the largest surrounding edge, as shown in Figure 3(c). The holes in the largest surrounding edge were filled to obtain the total number of pixels of the cerebral tissues and other components such as the skull. A threshold value of the Euclidean distance of possible tumors from the skull was determined based on the total number of brain pixels. The threshold value is directly proportional to the brain size.

**Figure 4:**
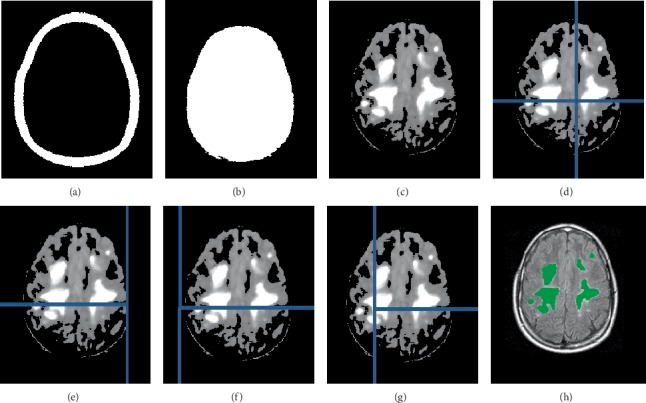
Morphological operations on MRI image and detection of tumors, (a) The largest surrounding edge expanded inward, (b) the largest remaining connected component, (c) The modified MRI image, (d) splitting the MRI image into 200 × 200 patches, (e–g) Shifting the horizontal indices before repeating the detection process (h) The resultant detected tumors.

The next step was applying morphological operations on the original MRI image in order to detect the tumors. The first operation was applying a binary mask that considers the *k*-means cluster with the medium intensity regions and the largest surrounding edge that was expanded towards the centre by morphological erosion (Figure 4(a)) using the following binary kernel:

(3) $$\begin{array}{c} \begin{array}{ccc} 0 & 1 & 0\\ 1 & 1 & 1\\ 0 & 1 & 0 \end{array}. \end{array}$$

The expanded largest edge was then inverted and multiplied by the medium intensity *k*-means cluster and the largest connected component was considered for hole filling as shown in Figure 4(b). The second operation was filtering the original MRI image by a small averaging filter to remove the noise then multiplying the filtered image by the original MRI image that had an adjusted intensity between 49.99% and 50.01% in order to focus on pixels that represent abnormal tissues. The contrast of the resultant image was further adjusted between 10% and 90% for greater concentration on abnormal tissues. Finally, both modified images were multiplied with each other to give the image in Figure 4(c).

After obtaining the modified MRI image, the next step was splitting it into small patches in order to detect small size tumors, as shown in Figure 4(d). Each patch has an initial size of 200 × 200 pixels, which was scaled up to three times larger for processing. Each enlarged patch was first smoothed using an averaging filter followed by morphological operations which include; low intensity elimination; conversion to multiple binary images using threshold values of 0.1, 0.3, 0.5, 0.7, 0.9, and Otsu's threshold value [22]; elimination of small objects; filling holes; object counting; continuous erosion using an incremental disk until the number of binary objects was equal to one object; and region growing using the centre of the detected binary object as the seed point [23]. These operations were repeated with an additional initial step of intensity adjustment between 30% and 70% in order to increase the contrast of all possible tumors and eliminate false dark regions. The processed images were added together to give a final image that contained regions of possible tumors.

**Figure 5:**
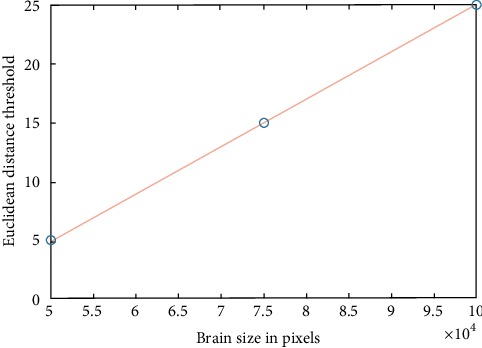
Euclidean distance threshold value with respect to the brain size.

The whole process that started with splitting the image into patches was repeated in steps of 50 pixels l shift within each patch as shown in Figures 4(e)–4(g). The shifting and processing produced output images with regions that had pixel values between zero and four. Only regions with pixel values between zero and two were considered as possible tumors while others were ignored. The last step was evaluating the detected tumors to determine if they were true tumors or not. The evaluation was performed by finding the Euclidean distance between each detected tumor and the largest surrounding edge of the skull. The possible tumor was discarded if its Euclidean distance from the skull was less than the selected threshold value which was determined based on the area of the brain image as shown in Figure 5. This relationship shows that the Euclidean distance threshold values are directly proportional to the total number of pixels in the brain image. Smaller threshold values such as 5 and 6 are assigned to smaller brain areas such as 5000 or 8000 pixels. A larger threshold value such as 25 is assigned to larger brain area such as 100000 pixels. The detected true tumors in the MRI image after applying tumor evaluation are shown in Figure 4(h). It can be noted that both small tumors and large tumors were detected while the surrounding false regions that are part of the skull were discarded.

**Figure 6:**
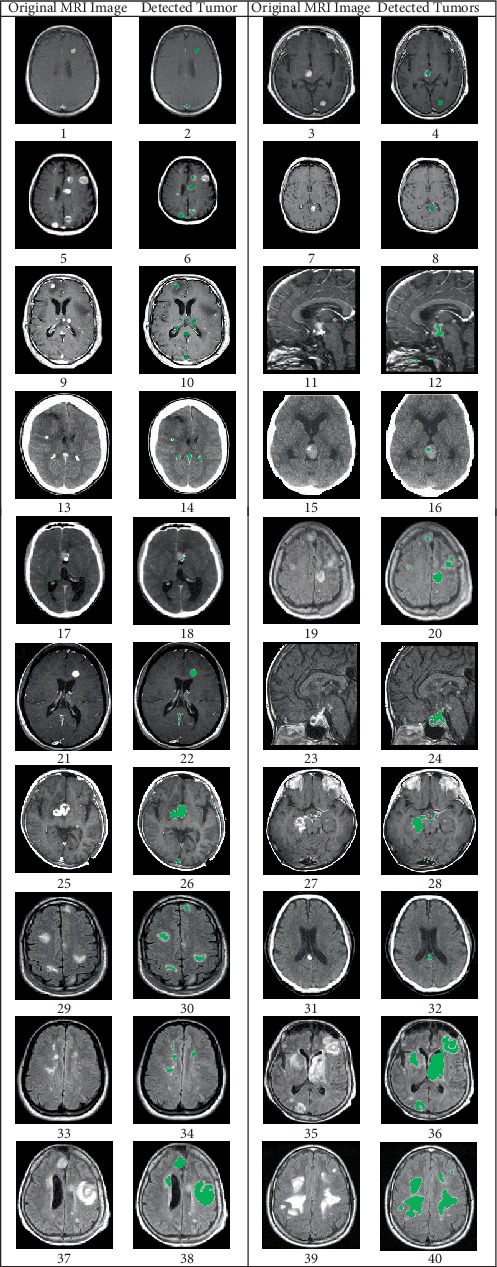
MRI brain images that contain tumors with different sizes and intensity levels and the detected tumors in each image.

The technique was also tested further on twenty MRI images that contained multiple tumors of different sizes as shown in Figure 6.

## 5. Discussion

Based on the implementation and testing results, the proposed technique is capable of detecting multiple large as well as small and low intensity tumors in MRI images. This is very evident in the original MRI image shown in Figure 6 (9) which has eight different size tumors and the detection results shown in Figure 6 (10) with all eight tumors detected and localized.

The performance of the proposed technique was further evaluated using different measurement parameters. The evaluation parameters used were Precision, which measures the amount of true positives and false positives; Recall which refers to completeness or sensitivity and measures the number of true positive and false negatives; Specificity which measures the true negative rate; Dice Score Coefficient which measures the overlap between manual and automatic segmentation; and Accuracy, which measures the rate of true positives and true negatives. These parameters were calculated using the following equations.

(4) $$\begin{array}{c} \text{Precision}=\frac{TP}{TP+FP} \end{array}$$

(5) $$\begin{array}{c} \text{Recall}=\frac{TP}{TP+FN} \end{array}$$

(6) $$\begin{array}{c} \text{Specificity}=\frac{TN}{TN+FP} \end{array}$$

(7) $$\begin{array}{c} \text{Dice Score Coefficient}=\frac{2TP}{2TP+FP+FN} \end{array}$$

(8) $$\begin{array}{c} \text{Accuracy}=\frac{TP+TN}{TP+FP+FN+TN}. \end{array}$$

In these equations, TP is the number of true positive pixels which indicates how many actual tumorous tissues have been correctly detected; FP is the number of false positive pixels which indicates how many pixels falsely detected as tumorous; FN is the number of false negative pixels, which refers to the pixels falsely detected as nontumorous; and TN is the number of true negative pixels and refers to the pixels correctly detected as nontumorous as the ground truth nontumorous pixels. The performance parameters were calculated using the detection results related to the MRI images shown in Figure 6. It can be noted from Table 1 that the average values for Precision, Accuracy, and Specificity are 98.48, 99.81, and 99.99% respectively. The Dice Score Coefficient average value is 95.81%, while the Recall average value is 92.16%.

The quantitative performance parameters of the proposed technique were compared with other tumor detection techniques proposed in previous research articles as shown in Table 2. The average value for Specificity is 99.99%, which is close to the maximum possible value. The Sensitivity is 92.16%, which is greater than the highest sensitivity value of 91.66% reported by Gujar et al. [19]. The Precision value is very close to the highest Precision value reported by Gujar et al. [19]. The Dice Score Coefficient is 95.81%, which is greater than the value of 80% reported by Hanuman et al. [16]. The Accuracy value is 99.81%, which is greater than the 92.62% value reported by Kullayamma et al. [21].

## 6. Conclusion

Effective MRI image segmentation is an essential step for the diagnosis and treatment of brain tumors. Detection of cancerous tumors at the very early stages of their development enables doctors to determine the appropriate treatment and hence it enhances the patients' chances of full recovery. In this paper, an automated technique for the detection and localization of early-stage brain tumors in MRI images was implemented using a combination of *k*-means clustering, patch-based processing, object counting, and tumor evaluation. The technique was tested and implemented using twenty real brain MRI images, which were diagnosed by clinicians. In addition to large tumors, the proposed technique was able to detect early-stage tumors in MRI images regardless of their size, intensity variation, and location. In order to make the technique more robust, an adaptive approach for the patch scaling will be explored in the future.

## Figures and Tables

**Table 1:** Performance parameters of the proposed technique.

MRI image #	Detected pixels	Actual pixels	Precision	Recall	Specificity	Dice score coefficient	Accuracy
1	346	400	1.0000	0.8700	1.0000	0.9351	0.9996
2	472	571	1.0000	0.8266	1.0000	0.9051	0.9999
3	398	430	1.0000	0.9256	1.0000	0.9614	0.9988
4	107	110	1.0000	0.9727	1.0000	0.9862	0.9999
5	1539	1619	1.0000	0.9922	1.0000	0.9961	0.9999
6	1726	1532	0.6964	0.8051	0.9975	0.8920	0.9961
7	217	240	1.0000	0.9042	1.0000	0.9497	0.9994
8	62	63	1.0000	0.9841	1.0000	0.9920	1.0000
9	24	30	1.0000	0.8000	1.0000	0.8889	0.9997
10	510	519	1.0000	0.9827	1.0000	0.9913	0.9997
11	1859	2056	1.0000	0.9042	1.0000	0.9497	0.9993
12	2331	14	1.0000	0.9940	1.0000	0.9970	0.9999
13	4195	4320	1.0000	0.9711	1.0000	0.9853	0.9993
14	3057	3257	1.0000	0.9386	1.0000	0.9683	0.9989
15	518	582	1.0000	0.8900	1.0000	0.9418	0.9982
16	107	114	1.0000	0.9386	1.0000	0.9683	0.9999
17	509	591	1.0000	0.8613	1.0000	0.9255	0.9986
18	2869	3259	1.0000	0.8803	1.0000	0.9364	0.9885
19	1809	2161	1.0000	0.8371	1.0000	0.9113	0.9884
20	2167	2217	1.0000	0.9774	1.0000	0.9886	0.9980
*Average*	–	–	0.9848	0.9216	0.9999	0.9581	0.9981

**Table 2:** Comparison between the proposed technique and other techniques.

Evaluation parameter	Techniques			
	Kullayamma et al. [21]	Gujar et al. [19]	Hanuman et al. [16]	Proposed technique
Precision	–	1.0000	0.9000	0.9848
Sensitivity	0.8402	0.9166	0.7000	0.9216
Specificity	–	–	1.0000	0.9999
Dice Score Coeff.	–	–	0.8000	0.9581
Accuracy	0.9262	0.8967	–	0.9981

## Data Availability

The data used to support the findings of this study are included within the article.
